# Development and validation of a nomogram for predicting axillary reverse mapping lymph node metastasis in breast cancer patients

**DOI:** 10.3389/fonc.2026.1854483

**Published:** 2026-07-21

**Authors:** Xiaoli Kong, Fanchao Xu, Yiran Liang, Xiaoyan Li, Tingting Ma, Liyu Jiang, Qifeng Yang

**Affiliations:** 1 Department of Breast Surgery, General Surgery, Qilu Hospital of Shandong University, Jinan, Shandong, China; 2 Biological Resource Center, Qilu Hospital of Shandong University, Jinan, Shandong, China; 3 Research Institute of Breast Cancer, Shandong University, Jinan, Shandong, China

**Keywords:** ARM lymph node, axillary lymph node dissection, axillary reverse mapping, breast cancer, nomogram, sentinel lymph node

## Abstract

**Background:**

Axillary reverse mapping (ARM) aims to preserve upper limb lymphatic drainage during breast cancer surgery to reduce lymphedema risk, but the metastatic potential of ARM lymph nodes and its preoperative predictors remain unclear.

**Methods:**

A retrospective analysis was conducted on a development cohort of 142 breast cancer patients, with an independent temporal validation cohort of 54 consecutive patients. Indocyanine green and methylene blue were used to map upper limb and breast lymphatic drainage, respectively. Identified ARM lymph nodes were preserved and pathologically examined. Independent predictors of metastasis were identified via univariable and multivariable logistic regression. A preoperative nomogram was developed and validated.

**Results:**

ARM lymph nodes were successfully identified in 142 (92.81%) patients, with metastasis in 33 (23.24%). Four lymphatic drainage patterns were defined based on ARM-sentinel lymph node distance and shared trunks. Multivariate analysis identified several independent predictors: menarche age ≤ 12 years (P = 0.012), elevated fibrinogen (P = 0.008), elevated CA15-3 (P = 0.015), elevated platelet-to-lymphocyte ratio (P = 0.021), presence of sentinel ARM lymph nodes (P = 0.003), larger ARM lymph node long diameter (P < 0.001), and ultrasound-detected axillary abnormalities (P = 0.007). The nomogram demonstrated excellent discrimination in the development cohort (AUC = 0.934) and acceptable discrimination in the temporal validation cohort (AUC = 0.826).

**Conclusions:**

ARM lymph node metastasis occurs in nearly one-quarter of patients. Our preoperative nomogram, incorporating menarche age, inflammatory and tumor markers, imaging, and ARM anatomy, can predict ARM lymph node metastasis risk, supporting selective ARM node preservation and reducing breast cancer-related lymphedema without compromising oncologic safety.

## 1 Introduction

Breast cancer (BC) remains the most commonly diagnosed malignancy and the leading cause of cancer-related morbidity among women worldwide (1). For patients with early-stage BC, surgery is the cornerstone of treatment. Axillary lymph node dissection (ALND) has long been the standard surgical approach for axillary staging and regional control. However, ALND is associated with a substantial risk of postoperative complications, among which upper extremity lymphedema is the most debilitating, not only impairing physical function but also adversely affecting psychological well-being and quality of life (2). Since the late 1990s, sentinel lymph node biopsy (SLNB) has been widely adopted as a less invasive alternative to ALND, which significantly lowers the risk of breast cancer-related lymphedema (BCRL) by preserving non-SLNs and surrounding tissue (3–5). Nevertheless, lymphedema remains a concern even in patients receiving SLNB (6). The ACOSOG Z0011 trial reported a 2% incidence (6 of 268) of subjective lymphedema at one year following sentinel lymph node dissection (SLND) alone (7). Similarly, a 2013 study by De Groef et al. observed an 8% incidence (8/100) of upper limb lymphedema one year after SLNB (8). These findings underscore the persistent risk of BCRL even with minimally invasive techniques, highlighting the need for novel strategies to further preserve lymphatic integrity and improve long-term outcomes.

Axillary reverse mapping (ARM) has emerged as a promising technique to protect the lymphatic drainage of the upper limb during axillary surgery. Pioneering work by Hama et al. using two-color spectral fluorescence lymphangiography demonstrated the anatomical distinction between the lymphatic pathways of the breast and the upper extremity (9), providing the foundation for ARM. Subsequent studies confirmed the feasibility of ARM in axillary surgery for BC patients (10, 11). Notably, randomized controlled trials (RCTs) have shown that identification and preservation of ARM lymph nodes significantly reduce the incidence of BCRL (5, 12, 13). Despite these advantages, a critical concern persists: the potential for ARM lymph nodes to harbor metastases. In the absence of reliable preoperative predictors, preserving ARM lymph nodes without pathological confirmation may raise oncological safety concerns, particularly in patients at higher risk of nodal involvement. Currently, there is a lack of robust clinical tools to assess the likelihood of ARM node metastasis, which limits the broader application of this protective technique. Therefore, identifying independent risk factors for ARM lymph node metastasis is essential.

**Figure 1:**
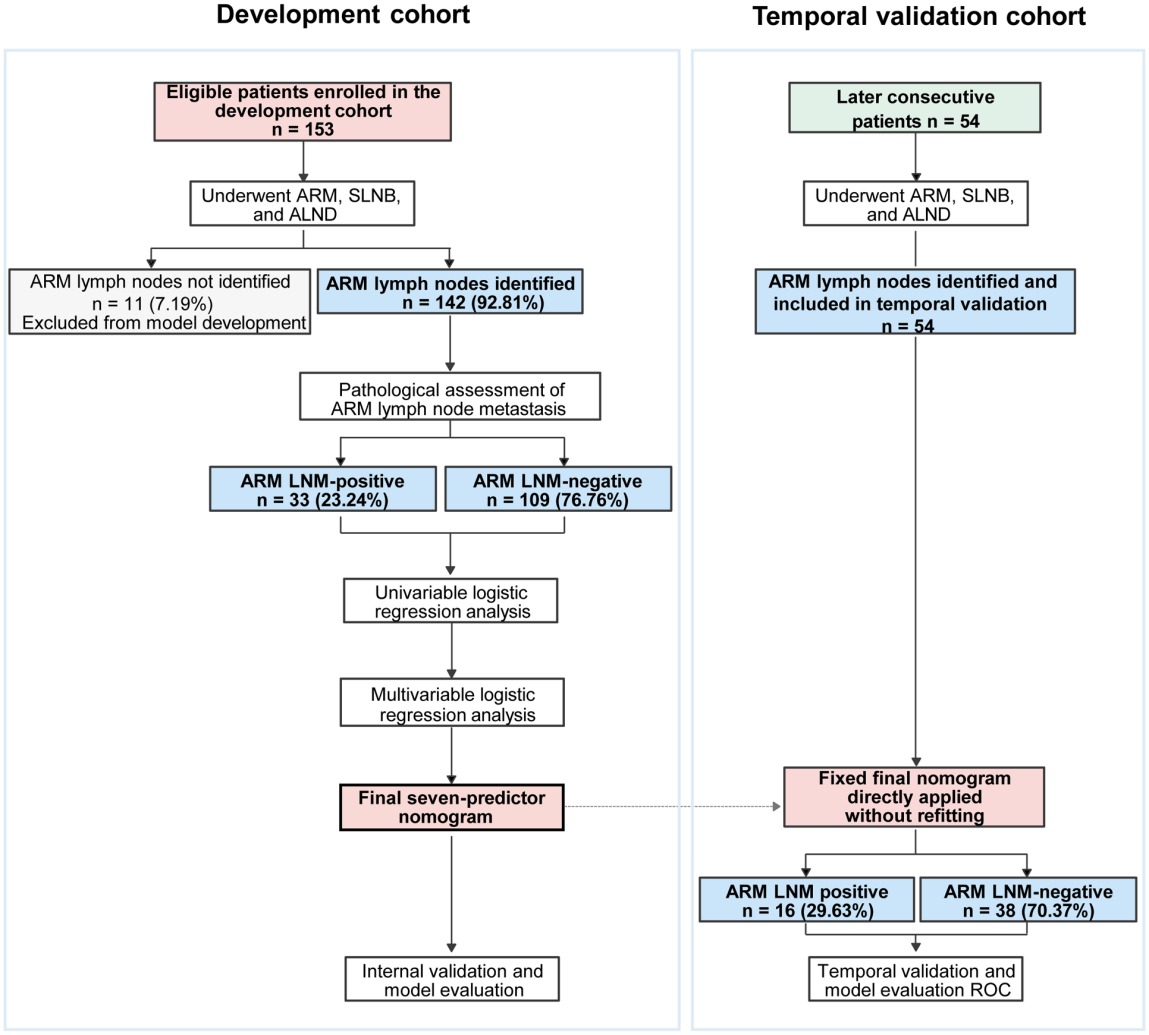
Flow diagram of the research program. A total of 153 patients in the development period underwent ARM, SLNB, and ALND. ARM lymph nodes were identified in 142 patients and were not identified in 11 patients, who were excluded from model development. Among the 142 patients included in the development cohort, 33 had ARM lymph node metastasis and 109 had no ARM lymph node metastasis. Univariable and multivariable logistic regression analyses were performed to identify predictors, and a seven-predictor nomogram was developed and internally evaluated using ROC curve analysis, calibration analysis, and decision curve analysis. The fixed final nomogram was then directly applied to an independent temporally separated cohort of 54 later consecutive patients without refitting, coefficient re-estimation, variable re-selection, or cutoff optimization. ARM, axillary reverse mapping; LN, lymph node; LNM, lymph node metastasis; SLNB, sentinel lymph node biopsy; ALND, axillary lymph node dissection; ROC, receiver operating characteristic; DCA, decision curve analysis.

In this study, we comprehensively analyzed the clinical characteristics, laboratory findings, and pathological data from a cohort of breast cancer patients to determine risk factors of ARM lymph node metastasis. On this basis, we developed and validated a nomogram to individualize the prediction of ARM lymph node metastasis. The study flowchart is presented in **Figure 1**. This tool aims to support clinical decision-making by balancing the benefits of lymphedema prevention with oncological safety, ultimately improving both survival outcomes and quality of life for breast cancer patients.

## 2 Methods

### 2.1 Patient selection

This study included a development cohort of 142 patients with stage I-III breast cancer who underwent surgery at Qilu Hospital of Shandong University between June 2023 and June 2024, and an independent temporal validation cohort of 54 consecutive patients treated between December 2024 and April 2025, after the development period. All patients in both cohorts underwent the same surgical procedures, including ARM, SLNB and ALND. The inclusion criteria were: (1) primary surgical treatment for breast cancer (excluding diagnostic biopsy); (2) pathologically confirmed invasive breast cancer without distant metastasis; (3) absence of rheumatologic, autoimmune, or hematologic disorders; (4) no prior radiotherapy to the breast or axilla; (5) completion of ALND with full availability of clinical and pathological data. The exclusion criteria included: (1) non-primary breast surgery; (2) non-invasive BC (e.g., DCIS) or BC with distant metastasis; (3) presence of rheumatologic or hematologic diseases; (4) prior breast or axillary irradiation; (5) not undergoing ALND or lacking complete clinical/pathological records. The study was approved by the Medical Ethics Committee of Qilu Hospital, Shandong University (Clinical Trial Registration: NCT02651142), and all participants provided written informed consent.

### 2.2 Reagents and instruments

The two tracers used during surgery, indocyanine green (ICG) and methylene blue (MB), were procured by Qilu Hospital of Shandong University. MB (1%, 20 mg/2 ml per vial) was produced by Jichuan Pharmaceutical Group Co., Ltd., and ICG (25 mg per vial) was manufactured by Dandong Yichuang Pharmaceutical Co., Ltd. The imaging system used was the M-2 near-infrared fluorescence imaging device (M.L.-station V1.0.0, Jinan Microvision Intelligent Technology Co., Ltd.), which features dedicated “ICG” and “MB” modes to enable the identification and localization of lymphatic pathways stained with ICG and MB, respectively.

### 2.3 Surgical procedure

**Figure 2:**
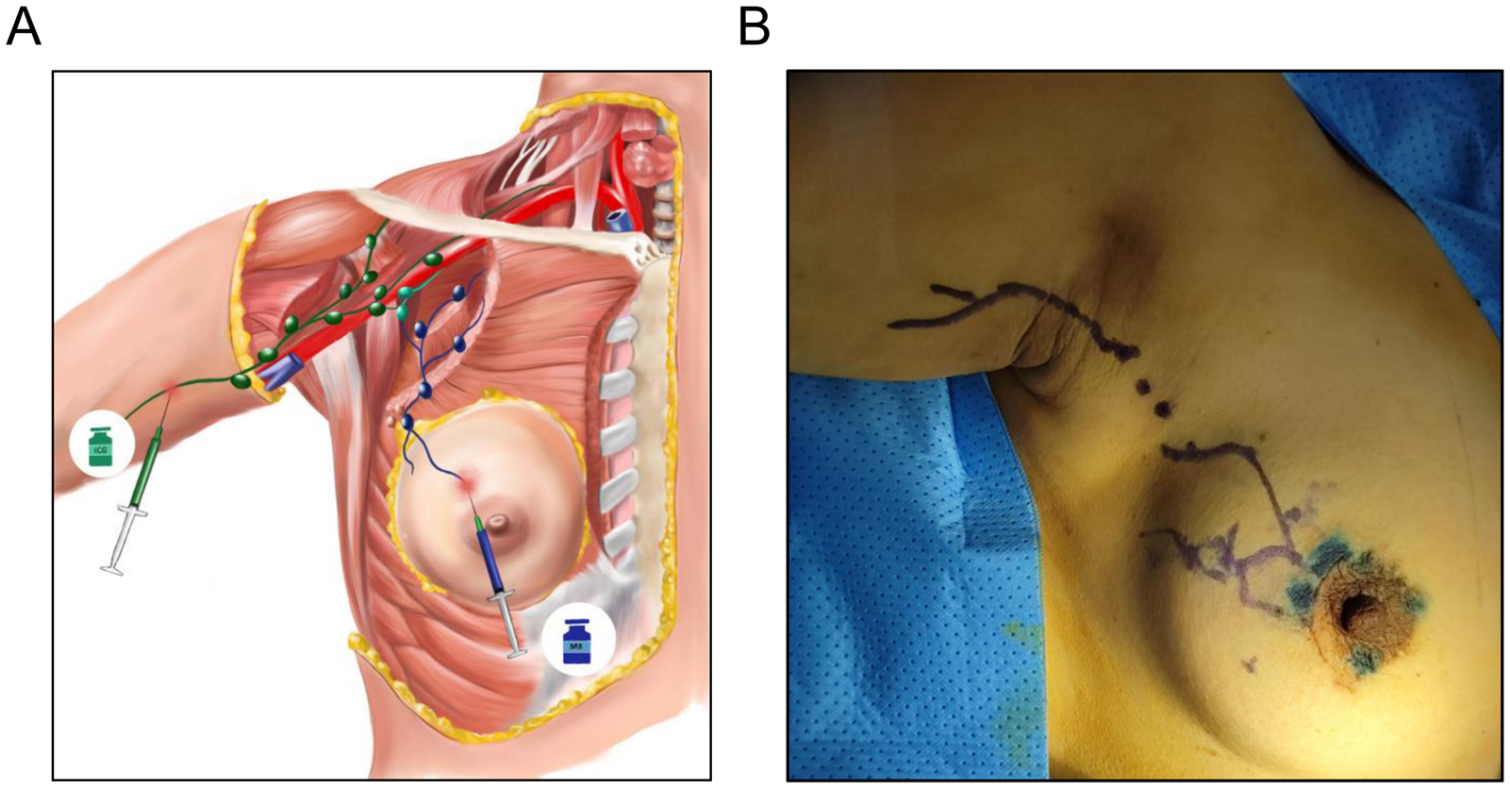
Dual-fluorescence tracing technology. **(A)** Diagram of surgical procedure. ICG was injected subcutaneously into the intermuscular groove of the upper arm to visualize upper-limb lymphatic drainage. MB was injected around the areola and tumor to visualize breast lymphatic drainage. The first lymph node stained blue is the sentinel lymph node. The flesh-colored lymph nodes adjacent to the sentinel lymph node are para-sentinel lymph nodes. The teal areas indicate the phenomenon of common stem. **(B)** The lymphatic drainage pathways of the breasts and upper limbs are depicted on the body surface.

In this study, MB was used to trace mammary lymphatic drainage, while ICG was employed to map lymphatic flow from the affected upper limb (**Figure 2**).

One hour prior to axillary surgery, 1 mL of ICG (0.05 mg/mL) was injected subcutaneously into the intermuscular groove of the upper arm. “ICG” mode of the near-infrared fluorescence imaging device was used to visualize the lymphatic vessel drainage pathways on the monitor in real-time. The course of the lymphatic vessels from the upper arm was then marked on the skin surface using a sterile surgical marking pen. During surgery, ARM lymph nodes were identified by tracing fluorescent lymphatic pathways using “ICG” mode, carefully preserved, and individually labeled for pathological examination.

Fifteen minutes before surgery, 1 mL of MB (10 mg/mL) was injected intradermally at the 0, 3, 6, and 9 o’clock positions around the areola and overlying the tumor. The near-infrared fluorescence imaging system was set to “MB” mode to enable real-time visualization of the lymphatic pathways on the monitor. The course of the lymphatic vessels from breast was similarly outlined on the skin using a sterile surgical marking pen. During surgery, blue-stained lymphatic vessels were identified under direct vision, and stained SLNs were traced, confirming using “MB” mode, and excised for histopathological analysis.

### 2.4 Data collection

Comprehensive data were collected from electronic medical records and pathological reports, including: (1) patient characteristics: age, ethnicity, age at menarche, marital status, fertility status, body mass index (BMI), laboratory parameters, clinical stage, and preoperative ultrasound findings; (2) tumor characteristics: histological type, tumor grade, lateralization, tumor number, tumor size, skin involvement, lymphovascular invasion, immunohistochemical profile, and axillary lymph node metastasis; (3) intraoperative data: detection rates of SLNs and ARM lymph nodes, the longest length of ARM lymph nodes, and axillary tumor involvement.

### 2.5 Histological assessment

All surgical specimens were fixed in formalin and stained with hematoxylin and eosin (H&E) following the standard protocols. Two experienced pathologists, blinded to patient information, independently evaluated each tissue section using a light microscope for histopathological assessment. Any discrepancies were resolved through consensus discussion to ensure diagnostic accuracy.

### 2.6 Risk factors screening and nomogram construction

Univariable logistic regression analysis was performed with patient and tumor characteristics as independent variables and the presence of metastasis in ARM lymph node as the dependent variable. Variables with *P* < 0.05 and odds ratios (ORs) with 95% confidence intervals (CIs) excluding 1 were considered potential risk factors. Subsequently, these candidates were entered into a multivariable logistic regression model using a bidirectional stepwise regression approach based on the Akaike Information Criterion (AIC) to optimize model fit and minimize overfitting. The significant predictors identified in the multivariable analysis were used to construct a nomogram for predicting the probability of ARM lymph node metastasis. In the nomogram, each variable is assigned a point scale based on its relative contribution to the outcome. The total score, derived by summing individual variable scores, corresponds to the predicted probability of ARM lymph node metastasis.

### 2.7 Model evaluation

The predictive performance of the nomogram was further assessed using the following methods. The receiver operating characteristic (ROC) curve and area under the curve (AUC) were used to evaluate the validity and reliability of the model. An AUC > 0.7 was considered acceptable discrimination, with values closer to 1.0 indicating superior performance. The Hosmer-Lemeshow test was used to assess calibration (P > 0.05 indicating good fit), and calibration was visualized via a calibration plot generated from 1,000 bootstrap resamples to correct for over-optimism. Decision curve analysis (DCA) was employed to assess the clinical utility of the nomogram by quantifying the net benefit across a range of threshold probabilities.

In the development cohort, to address the limited number of ARM lymph node metastasis events and the consequent risk of overfitting, the events per variable (EPV) ratio was calculated. Firth’s penalized likelihood logistic regression was performed as a sensitivity analysis using the same seven predictors included in the final nomogram. Additionally, more parsimonious models were explored using AIC- and BIC-based stepwise selection; the discrimination and calibration performances of these candidate models were compared with the original seven-predictor model using the AUC and Brier score. Multicollinearity among predictors was assessed using variance inflation factors (VIFs), with a VIF > 5 considered indicative of potential multicollinearity. Pairwise Spearman correlation analysis was also performed among continuous predictors.

### 2.8 Temporal validation

An independent temporally separated cohort of 54 consecutive patients treated after the development cohort was used for temporal validation. These patients underwent the same surgical procedures. The final seven-predictor model derived from the development cohort was fixed and directly applied to the temporal validation cohort without any variable selection, coefficient re-estimation, model refitting, or recalibration. The cutoff probability determined in the development cohort was applied unchanged in the validation cohort.

Model performance in the temporal validation cohort was assessed using the AUC, sensitivity, specificity, accuracy, positive predictive value, negative predictive value, and Brier score. Calibration was evaluated by calibration plots, calibration intercept, calibration slope, and observed-to-expected ratio. Decision curve analysis was also performed. Baseline characteristics between the development and temporal validation cohorts were compared to assess case-mix heterogeneity.

### 2.9 Statistical analysis

Statistical analyses were performed using SPSS (version 29.0.1, IBM Corp., Armonk, NY, USA) and R (version 4.4.2, R Foundation for Statistical Computing, https://www.R-project.org). Normally distributed continuous variables were presented as mean ± standard deviation (SD) and compared using independent samples t-tests. Non-normally distributed variables were reported as median (interquartile range, IQR) and analyzed using the Mann-Whitney U test. Categorical variables were expressed as frequencies (percentages) and compared using the Pearson chi-square test or Fisher’s exact test, as appropriate. All tests were two-sided, and statistical significance was defined as P < 0.05.

## 3 Results

### 3.1 General characteristics of breast cancer patients

A total of 153 patients in the development period who met the inclusion criteria underwent ARM, followed by MB-guided SLNB and completion ALND. ARM lymph nodes were successfully identified in 142 patients, resulting in a detection rate of 92.81% (142/153) for the ARM technique. The remaining 11 patients showed no identifiable ARM nodes and were excluded from further analysis. The patients’ ages ranged from 28 to 81 years, with a mean age of 53.06 ± 10.83 years, and the mean BMI was 24.71 ± 3.29 kg/m². Among the 142 patients, 33 (23.24%) had metastasis in ARM lymph nodes (ARM LNM-positive), while 109 (76.76%) were negative (ARM LNM-negative).

Clinical and pathological characteristics were compared between the ARM LNM-positive and ARM LNM-negative groups to identify potential risk factors for ARM lymph node metastasis (**Table 1**, **Supplementary Table 1**). Statistically significant differences were observed in the following variables: fibrinogen level, platelet-to-lymphocyte ratio (PLR), carbohydrate antigen 15-3 (CA15-3), nipple inversion, clinical stage, tumor size (longest diameter), lymphovascular invasion (LVI), pathological stage, ER/PR expression status, and ultrasound-detected lymph node abnormalities. CEA and CA125 showed no significant differences between groups (P = 0.748 and P = 0.868, respectively). No significant differences were found in age, BMI, tumor quadrant, histological grade, menopausal status, or other clinical variables.

**Table 1:** Baseline characteristics of breast cancer patients stratified by ARM lymph node metastasis status.

Characteristics	ARM LNM negative group (n = 109)	ARM LNM positive group (n = 33)	*P*
Age (years), Mean ± SD	53.110 ± 10.694	52.909 ± 11.441	0.926
BMI (kg/m2), Mean ± SD	24.598 ± 3.250	25.068 ± 3.428	0.473
Menarche age group, n (%)			0.002
≤.0	10 (9.174)	11 (33.333)	
>12	99 (90.826)	22 (66.667)	
Family history breast cancer, n (%)			0.741
No	100 (91.743)	29 (87.879)	
Yes	9 (8.257)	4 (12.121)	
Tumor lateralization, n (%)			0.099
Left	64 (58.716)	14 (42.424)	
Right	45 (41.284)	19 (57.576)	
Tumor quadrant, n (%)			0.318
Upper outer	44 (40.367)	19 (57.576)	
Upper inner	30 (27.523)	5 (15.152)	
Lower inner	7 (6.422)	3 (9.091)	
Lower outer	13 (11.927)	4 (12.121)	
Areola area	15 (13.761)	2 (6.061)	
Clinical stage, n (%)			0.016
I	29 (26.606)	2 (6.061)	
II	72 (66.055)	25 (75.758)	
III	8 (7.339)	6 (18.182)	
Fibrinogen, Mean ± SD	2.883 ± 0.463	3.115 ± 0.389	**0.010**
PLR, Mean ± SD	140.424 ± 51.437	170.302 ± 59.347	**0.006**
CEA (ng/ml), M (Q_1_, Q_3_)	1.560 (1.030, 2.250)	1.410 (0.970, 2.040)	0.748
CA15-3 (U/mL), M (Q_1_, Q_3_)	9.180 (6.420, 12.400)	14.820 (12.300, 18.600)	**<.001**
CA125 (U/mL), M (Q_1_, Q_3_)	10.200 (7.330, 14.100)	9.700 (7.480, 15.900)	0.868
Pathological stage, n (%)			<.001
I	30 (27.523)	0 (0.00)	
II	59 (54.128)	10 (30.303)	
III	20 (18.349)	23 (69.697)	
Vascular tumor thrombus, n (%)			<.001
No	83 (76.147)	14 (42.424)	
Yes	26 (23.853)	19 (57.576)	
Ultrasound Abnormal lymph nodes, n (%)			<.001
No	70 (64.220)	9 (27.273)	
Yes	39 (35.780)	24 (72.727)	

SD, standard deviation; M, Median; Q_1_, 1st Quartile; Q_3_, 3rd Quartile; BMI, Body Mass Index; RBC, Red Blood Cell Count; HB, Hemoglobin; WBC, White Blood Cell Count; PLR, Platelet-to-Lymphocyte Ratio; NLR, Neutrophil-to-Lymphocyte Ratio; CEA, Carcinoembryonic Antigen; CA153, Cancer Antigen 15-3; CA125, Cancer Antigen 125. Bold values, P < 0.05.

### 3.2 Distribution patterns of ARM lymph nodes

Based on our previously established theory of functional lymphatic anatomy (14, 15), we defined the concept of SLN according to its position within the lymphatic drainage pathways. Specifically, the first blue-stained lymph node identified along the lymphatic channel draining from the breast is defined as the SLN. Lymph nodes directly connected to the efferent lymphatic vessel of the SLN are designated as post-SLNs. The enlarged lymph nodes located adjacent to the SLN but not stained with MB are classified as para-SLNs. Additionally, lymph nodes exhibiting fluorescence under “ICG” mode are identified as ICG-labeled ARM lymph nodes. If an ARM lymph node appears blue to the naked eye or show fluorescence under “MB” mode, it is considered a “blue-stained ARM node”. When an ARM node coincides with the SLN, it is termed a sentinel ARM lymph node; if it corresponds to a post-SLN, it is labeled a post-sentinel ARM lymph node (**Supplementary Figure 1**).

SLNs were successfully detected in all 142 patients, with a total of 297 nodes retrieved (mean: 2.09 per patient). Among them, SLN metastasis was detected in 76 patients. Additionally, para-SLNs were identified in 32 patients, and post-SLNs in 63 patients. A total of 486 ARM lymph nodes were identified, averaging 3.42 per patient. In the 33 patients with ARM node metastasis, a total of 71 ARM nodes were involved. Notably, 28 patients (19.72%) had at least one lymph node that functioned as both a SLN and an ARM node (sentinel ARM lymph nodes). Furthermore, 55 patients (38.73%) exhibited MB staining of ARM nodes, indicating a “common stem” lymphatic drainage pattern. Detailed characteristics of lymph node distribution according to metastasis status of ARM lymph node are summarized in **Supplementary Table 2**.

During surgery, the shortest distance (D, cm) between the ARM lymph node and the SLN in a natural, non-stretched state was recorded to investigate the distribution pattern of ARM lymph nodes relative to the SLN. Among the 142 patients, the SLN coincided with an ARM lymph node (D = 0 cm) in 28 cases (19.72%). In 2 cases (1.41%), ARM lymph nodes were located within 1 cm of the SLN; both were identified as post-SLNs. ARM lymph nodes were situated at distances >1 cm but ≤2 cm from the SLN in 15 cases (10.56%); >2 cm but ≤5 cm in 69 cases (48.59%); and >5 cm in 25 cases (17.61%) (**Supplementary Table 3**).

### 3.3 Lymphatic drainage patterns in the axilla

The above findings indicate that ARM lymph nodes are predominantly distributed within a 2–5 cm radius around the SLN.

**Figure 3:**
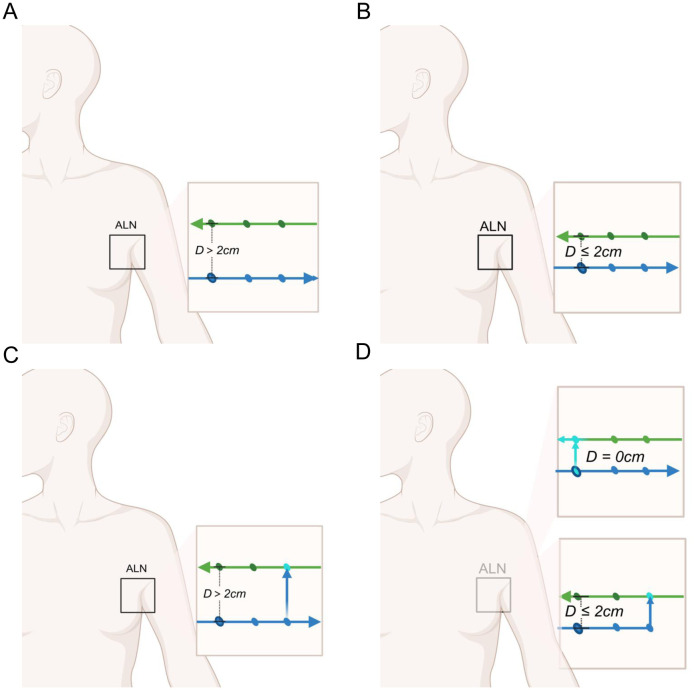
Schematic patterns of axillary lymphatic drainage. **(A–D)** represent the four types of distribution patterns based on the common lymphatic stem and the distance (D) from the sentinel lymph node (SLN): **(A)** no common stem and distant (D > 2 cm); **(B)** no common stem but close (D ≤ 2 cm); **(C)** common stem and distant (D > 2 cm); **(D)** common stem and close (D ≤ 2 cm). Green arrows indicate axillary lymphatic pathways, blue arrows indicate breast lymphatic pathways, and circles represent lymph nodes. ALN, axillary lymph nodes.

Using a 2 cm distance threshold to define proximity to the SLN, combined with the presence or absence of MB staining in ARM lymph nodes as an indicator of a shared lymphatic stem, the distribution patterns of breast and upper limb lymphatic drainage in the axillary region can be categorized into four types (**Figure 3**): (1) No common stem and distant type: ARM lymph nodes were not stained with MB and located >2 cm from the SLN (53.52% of patients); (2) No common stem but close type: ARM lymph nodes were not stained with MB but located ≤2 cm from the SLN (7.74%); (3) Common stem but distant type: ARM lymph nodes were MB-stained but located >2 cm from the SLN (16.20%); (4) Common stem and close type: ARM lymph nodes were MB-stained and located ≤2 cm from the SLN (22.54%) (**Supplementary Table 4**).

### 3.4 Predictors of ARM lymph node metastasis

To identify which factors could predict ARM lymph node metastasis before surgery, univariate logistic regression analysis was performed to explore potential predictors of ARM lymph node metastasis. Several risk factors were identified, including age at menarche ≤ 12 years, nipple inversion, advanced clinical stage, elevated fibrinogen, CA15-3, progesterone, or PLR, SLN metastasis, large ARM lymph node long diameter or pathological tumor long diameter, presence of sentinel ARM lymph nodes, low expression of ER/PR, and ultrasound-detected lymph node abnormalities (**Supplementary Table 5**). Multivariate logistic regression analysis further confirmed the following independent predictors of ARM lymph node metastasis: age at menarche ≤ 12 years, elevated fibrinogen/CA15-3/PLR, SLN metastasis, presence of sentinel ARM lymph nodes, larger ARM lymph node long diameter, and ultrasound-detected lymph node abnormalities. The corresponding ORs and P-values are presented in **Table 2**.

**Table 2:** Independent risk factors for axillary reverse mapping (ARM) lymph node metastasis in breast cancer patients identified by multivariate logistic regression analyses.

Variable	Multivariate logistic					
	Beta	SE	Z	OR	CI	P
Fibrinogen	2.347	0.938	2.503	10.458	1.965 ~ 121.082	**0.012**
CA15-3	0.129	0.052	2.472	1.138	1.027 ~ 1.261	**0.008**
PLR	0.020	0.009	2.291	1.020	1.003 ~ 1.038	**0.022**
ARM lymph node long diameter	1.778	0.629	2.828	5.918	1.726 ~ 20.291	**0.005**
Pathological tumor long diameter	1.610	0.583	2.760	5.003	1.595 ~ 15.698	0.700
Menarche age group						
≤12				1.000 (Reference)		
>12	-3.755	1.192	-3.151	0.023	0.002 ~ 0.242	**0.002**
Sentinel lymph node metastasis						
No				1.000 (Reference)		
Yes	4.491	1.330	3.377	89.211	6.583 ~ 1208.997	**<.001**
Sentinel ARM lymph node						
No				1.000 (Reference)		
Yes	2.831	1.108	2.556	16.964	1.935 ~ 148.745	**0.011**
Ultrasound Abnormal lymph nodes						
No				1.000 (Reference)		
Yes	2.091	0.944	2.214	8.092	1.271 ~ 51.516	**0.027**

CA15-3, Cancer Antigen 15-3; PLR, Platelet-to-Lymphocyte Ratio; ARM, Axillary Reverse Mapping; SE, Standard Error; Z, Z-Score; OR, Odds Ratio; CI, Confidence Interval; P, P-Value. Bold values, P < 0.05.

### 3.5 Development and validation of nomogram for ARM lymph node metastasis status

**Figure 4:**
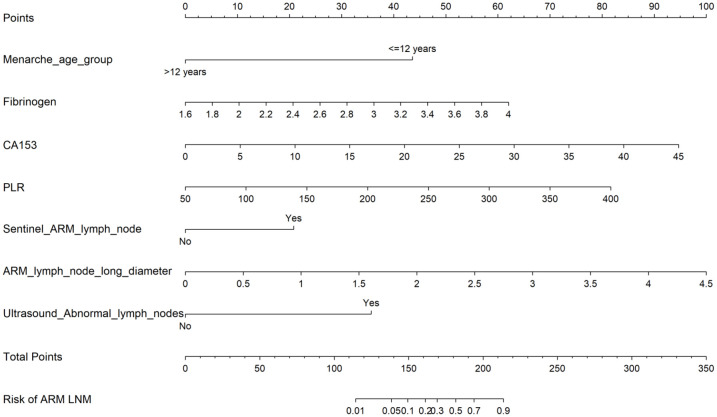
Nomogram for risk of ARM lymph node metastasis in patients with breast cancer. We used the “rms” package in R to construct a nomogram based on seven variables screened using univariable and multivariable logistic regression analyses. Each variable corresponds to a score on the top point axis. Add up the scores of each variable to obtain the total score. Project the total score onto the risk/probability axis to estimate the probability of ARM lymph node metastasis. CA15-3, cancer antigen 15-3; PLR, platelet-to-lymphocyte ratio; ARM, axillary reverse mapping.

Despite its known association with ARM lymph node involvement, SLN metastasis was excluded from the final model due to its intraoperative or postoperative nature, limiting its utility for preoperative decision-making. Therefore, a predictive nomogram was developed using the remaining independent risk factors to enable preoperative risk stratification (**Figure 4**).

In the nomogram, each predictor is assigned a point score based on its relative contribution to metastasis risk. Higher levels of fibrinogen, CA15-3, and PLR, as well as larger ARM lymph node diameter, were associated with incrementally higher scores. Menarche age ≤ 12 years scored 42 points, presence of sentinel ARM lymph nodes scored 23 points (absence: 0 points), and ultrasound-detected lymph node abnormalities scored 36 points. The total score corresponds to the predicted probability of ARM lymph node metastasis: a total score below 138 suggests a risk of ARM lymph node metastasis below 10%, whereas a total score exceeding 200 indicates a 90% risk of metastasis.

**Figure 5:**
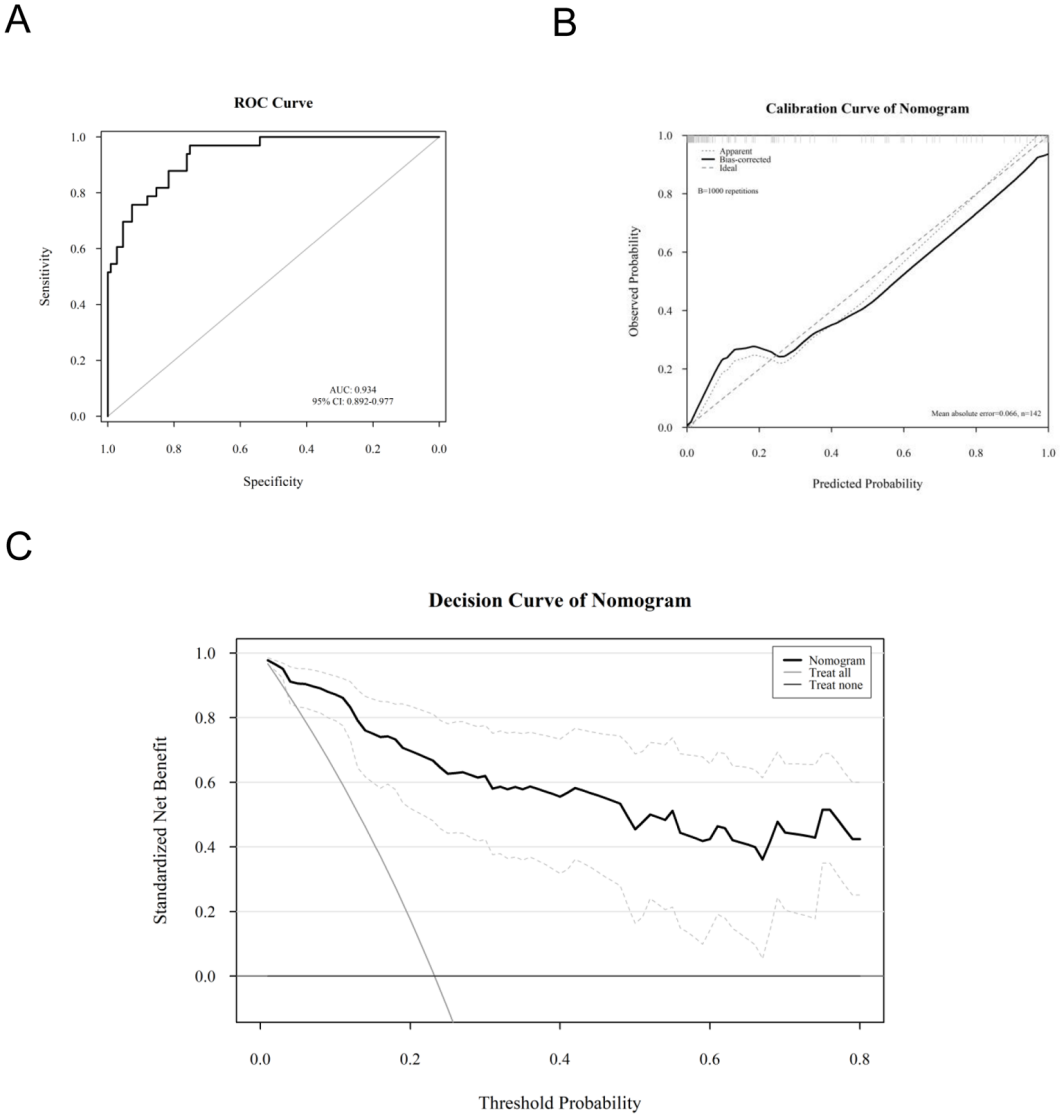
Performance evaluation of the nomogram model. **(A)** Receiver operating characteristic (ROC) curve of the nomogram, with an area under the curve (AUC) of 0.934, indicating high discriminative ability. **(B)** Calibration curve of the nomogram, showing agreement between predicted probability and observed outcomes. The solid line represents the apparent performance, the dashed line indicates bias-corrected performance after 1000 bootstrap resamples, and the dotted line represents the ideal reference line. AUC, the area under the curves. **(C)** Decision curve analysis (DCA) of the nomogram. The thick black line represents the net benefit of the nomogram, the gray oblique line represents the “treat-all” strategy, and the black horizontal line represents the “treat-none” strategy across a range of threshold probabilities. The light gray dashed lines indicate the bootstrap-based confidence interval of the nomogram net benefit. ROC, receiver operating characteristic; AUC, area under the curve; DCA, decision curve analysis.

The model demonstrated high discriminative ability in the development cohort, with an AUC of 0.934 (95% CI: 0.892-0.977) (**Figure 5A**). The bootstrap-corrected calibration curve showed acceptable agreement between predicted and observed probabilities of ARM lymph node metastasis, with a mean absolute error of 0.066 and a Brier score of 0.082 (**Figure 5B**). The Hosmer-Lemeshow test showed no evidence of poor fit (χ² = 3.795, df = 8, P = 0.875). Decision curve analysis (DCA) demonstrated a favorable net benefit across a wide range of threshold probabilities (**Figure 5C**).

**Figure 6:**
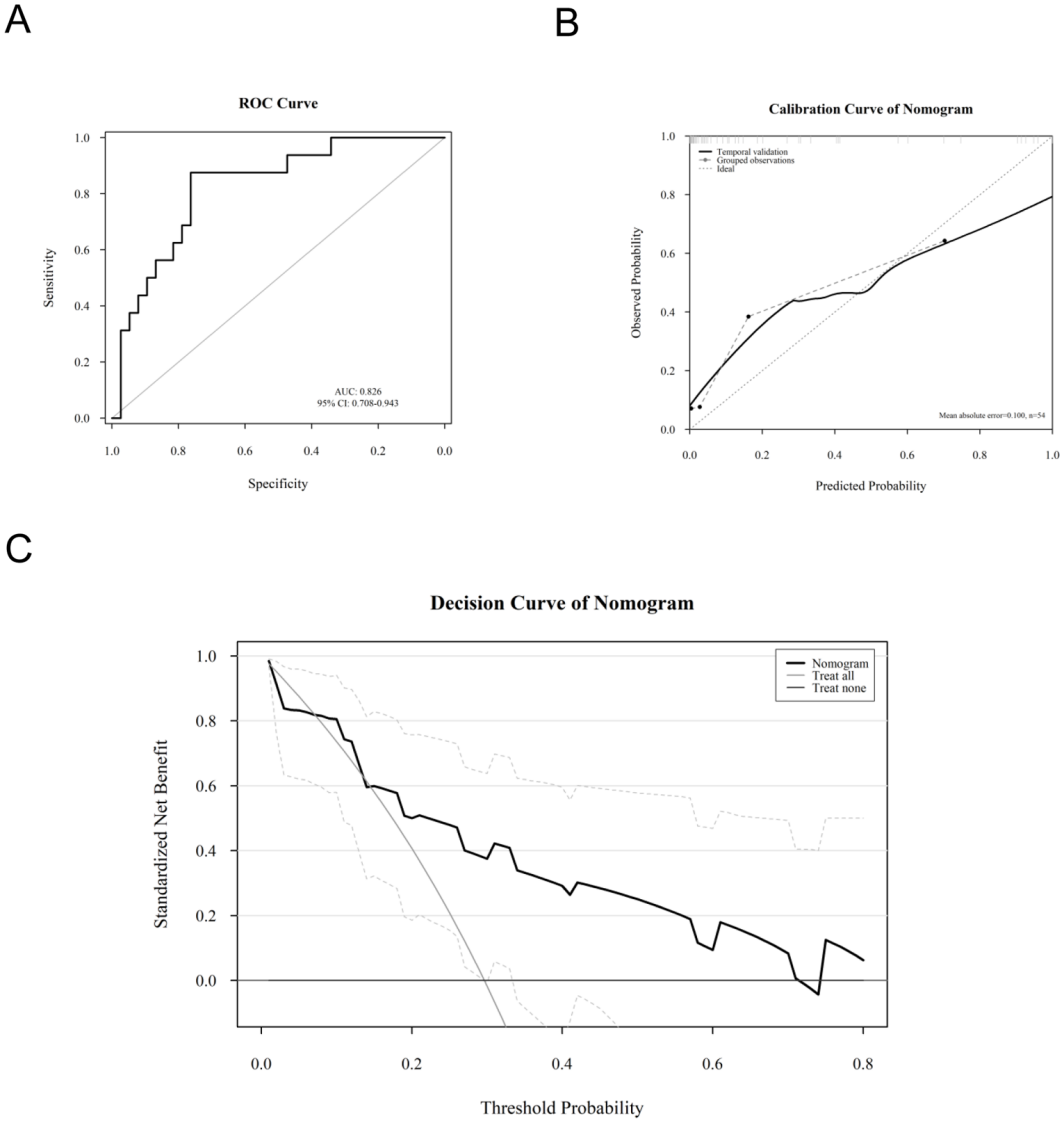
Temporal validation of the nomogram model. **(A)** Receiver operating characteristic (ROC) curve of the fixed nomogram model in the temporal validation cohort, with an area under the curve (AUC) of 0.826 and a 95% confidence interval of 0.708-0.943, indicating good discriminative ability. **(B)** Calibration curve of the nomogram in the temporal validation cohort, showing the agreement between predicted probability and observed outcomes. The solid line represents the calibration curve in the temporal validation cohort, the points indicate grouped observations, and the dotted line represents the ideal reference line. The mean absolute error was 0.100. **(C)** Decision curve analysis (DCA) of the nomogram in the temporal validation cohort. The thick black line represents the net benefit of the nomogram, the gray oblique line represents the “treat-all” strategy, and the black horizontal line represents the “treat-none” strategy across a range of threshold probabilities. The light gray dashed lines indicate the bootstrap-based confidence interval of the nomogram net benefit. ROC, receiver operating characteristic; AUC, area under the curve; DCA, decision curve analysis.

To further assess the generalizability of the model, an exploratory temporal validation was performed using 54 later consecutive patients who were not included in the development cohort. Among them, 16 patients had ARM lymph node metastasis and 38 did not, with an event rate of 29.6% (**Supplementary Table 6**). Baseline characteristics were largely comparable between the two cohorts, except that patients in the temporal validation cohort were slightly younger [49.00 (40.25-56.75) vs. 53.00 (47.00-60.00), P = 0.023] (**Supplementary Table 6**). The fixed seven-predictor nomogram was directly applied to the temporal validation cohort without any model refitting or cutoff optimization. The model achieved an AUC of 0.826 (95% CI: 0.708-0.943) (**Figure 6A**). Using the development-cohort cutoff of 0.115, the sensitivity, specificity, accuracy, positive predictive value, and negative predictive value were 0.812, 0.763, 0.778, 0.591, and 0.906, respectively. The Brier score was 0.168. The model predicted 12.351 events, whereas 16 events were observed, resulting in an observed-to-expected ratio of 1.295. The calibration intercept was 0.786 and the calibration slope was 0.327 (**Supplementary Table 7**), indicating suboptimal calibration in the temporal validation cohort (**Figure 6B**). Decision curve analysis showed that the nomogram still provided a positive net benefit across a range of clinically relevant threshold probabilities in the temporal validation cohort (**Figure 6C**).

### 3.6 Model robustness and sensitivity analyses

Considering the limited number of events in the development cohort, sensitivity analyses were performed to assess the robustness of the final model. Among the 142 patients, 33 had ARM lymph node metastasis, corresponding to an events-per-variable (EPV) ratio of 4.7 for the seven-predictor nomogram. Firth’s penalized logistic regression showed generally consistent directions of association with the conventional multivariable logistic regression model. Age at menarche >12 years remained inversely associated with ARM lymph node metastasis, while higher fibrinogen, CA15-3, PLR, larger ARM lymph node long diameter, and ultrasound-detected abnormal lymph nodes maintained positive associations. Sentinel ARM lymph node status also showed a positive direction of association but did not reach statistical significance after penalization (**Supplementary Table 8**). The predictive performance was comparable between the conventional and Firth penalized models, with AUC values of 0.934 and 0.935, respectively. The Brier score was 0.082 for both models, and the Hosmer-Lemeshow P values were 0.875 and 0.876, respectively.

In model simplification analyses, the AIC-selected model was identical to the original seven-predictor model. The BIC-selected model removed sentinel ARM lymph node status but showed no improvement in predictive performance (**Supplementary Table 9**). Therefore, the original seven-predictor nomogram was retained based on both predictive performance and clinical interpretability.

Multicollinearity assessment showed that all variance inflation factor (VIF) values were low, ranging from 1.049 to 1.133. Pairwise Spearman correlation analysis among continuous predictors also showed weak correlations, with a maximum absolute correlation coefficient of 0.169 (**Supplementary Table 10**). These results indicated no evidence of problematic multicollinearity.

## 4 Discussion

Axillary lymph node dissection (ALND) remains important for comprehensive assessment of axillary lymph node status, providing critical information for staging, prognosis prediction, and adjuvant therapy decisions. It also reduces the risk of locoregional recurrence, particularly in patients with nodal involvement. However, ALND is associated with various complications, most notably breast cancer-related lymphedema (BCRL), due to disruption of the upper extremity lymphatic drainage. Axillary reverse mapping (ARM) has emerged as a promising technique to preserve lymphatic channels from the upper limb, thereby reducing the incidence of BCRL. Nevertheless, concerns regarding the oncologic safety of ARM preservation persist, particularly in patients with potential ARM lymph node metastasis. In this study, ICG and MB were used to visualize the lymphatic vessel drainage pathways of the upper arm and breast, respectively. Among the 142 patients with identified ARM lymph nodes, 23.24% (33/142) exhibited ARM lymph node metastasis, while the majority (76.76%) were free of metastasis. This finding suggests that a substantial proportion of patients may be candidates for selective ARM node preservation, potentially avoiding unnecessary ALND and its associated complications.

To examine the spatial relationship between the upper limb lymphatic drainage pathways and the breast lymphatic drainage pathways, our study measured the shortest straight-line distance between the ARM lymph nodes and the SLN. The results showed that non-sentinel ARM lymph nodes are typically located at a relatively distant position from the SLN. Except for 28 cases where the SLN was concurrently an ARM lymph node (D = 0 cm), most ARM lymph nodes were distributed in the region where the distance from the SLN was greater than 2 cm and less than or equal to 5 cm. Previous studies have proposed designating the 2 cm region around the SLN as the SLN region (16). Non-SLN lymph nodes within this zone are also more likely to be resected. If a large number of ARM lymph nodes are present in this zone, the probability of their resection during surgery increases, thereby raising the risk of upper limb lymphedema postoperatively. Our study further classified axillary lymphatic drainage patterns into four types. Among these, the “No common stem and distant type” pattern had the highest proportion in this study (53.52%). This classification aids in more accurately assessing the potential risk of resecting ARM lymph nodes during surgery and guiding individualized lymphatic structure preservation strategies. However, due to individual anatomical variations, lymphatic patterns and SLN counts vary significantly among breast cancer patients. Previous studies (17, 18) have shown that the extent of SLN clearance and the number of SLNs detected vary among patients, and there may be a certain correlation between the number of SLNs detected and their false-negative rate (19). Therefore, surgeons are required to ensure that a sufficient number of SLNs are removed during breast cancer surgery to guarantee accurate tumor staging, while also minimizing damage to ARM lymph nodes to reduce the risk of postoperative upper limb lymphedema.

To ensure oncologic safety, accurate preoperative risk stratification is essential. To address this need, we first identified independent predictors of ARM lymph node metastasis through univariate and multivariate analyses. Notably, the clinically accessible predictors collectively reflect a combination of hormonal, inflammatory, tumor burden, anatomical, and imaging characteristics, offering a comprehensive risk assessment profile.

Early menarche has long been established as a risk factor for breast cancer development (20, 21), likely due to prolonged lifetime exposure to estrogen and associated molecular alterations in breast tissue. Orgéas et al. previously reported an association between early menarche and increased risk of axillary lymph node metastasis (22). Harris et al. further demonstrated that early menarche correlates with pro-oncogenic changes in both tumor and adjacent normal tissues (23). In the present study, we report for the first time that early menarche is an independent predictor of ARM lymph node metastasis, suggesting a potential role of early hormonal exposure in shaping lymphatic metastatic patterns. The underlying biological mechanisms, however, remain to be elucidated and warrant further investigation.

Fibrinogen, a key protein in coagulation and inflammation, has been increasingly recognized as a mediator of tumor progression. Elevated serum fibrinogen levels have been identified in multiple cancers (24–27), including breast cancer. Our findings confirm that higher fibrinogen levels correlate significantly with ARM node metastasis, which is consistent with a previous study that reported the link between elevated fibrinogen levels and poor prognosis of breast cancer patients (28). This phenomenon may be attributed to fibrinogen-mediated promotion of epithelial-mesenchymal transition (EMT), characterized by downregulation of adhesion junction protein E-cadherin and upregulation of mesenchymal marker vimentin (29), which enhance tumor cell motility and invasiveness.

The platelet-to-lymphocyte ratio (PLR) is derived from routine blood counts and serves as a non-specific marker of systemic inflammation (30). Accumulating evidence indicates that a pro-inflammatory microenvironment plays a pivotal role in tumor initiation, progression, and metastasis (31–33). This process may arise from an imbalance between anti-tumor immune responses and chronic inflammation, leading to elevated production of cancer-associated inflammatory mediators by tumor and stromal cells (34), such as TNF-α, IL-3, and IL-6. These cytokines can promote thrombocytosis and lymphopenia, resulting in an increased PLR. Given that both primary breast tumors and axillary lymph node metastases may be influenced by the host inflammatory and immune milieu, systemic inflammation markers like PLR may reflect tumor aggressiveness. Indeed, prior studies have linked elevated PLR to axillary lymph node metastasis in breast cancer (35), and our findings further extend this association specifically to ARM lymph node involvement, reinforcing the role of host inflammatory response in metastatic spread. Collectively, these results underscore the potential value of PLR as a readily available predictor of ARM node metastasis in breast cancer patients.

CA15–3 is a transmembrane glycoprotein that has been widely used as a serum tumor biomarker in breast cancer since the 1980s (36). Numerous studies have demonstrated its utility in the early detection of breast cancer progression and metastasis (37–39). Elevated serum CA15–3 levels are associated with higher tumor burden and axillary lymph node involvement (40, 41). In our nomogram, elevated CA15–3 was a significant predictor of ARM lymph node metastasis, supporting its utility not only in monitoring disease progression but also in preoperative risk assessment. This finding suggests that preoperative CA15–3 levels may serve as a valuable tool for risk stratification, aiding in the identification of patients at higher risk for ARM lymph node involvement and supporting personalized surgical decision-making. In our primary model, CA15–3 was treated as a continuous variable. Its functional form was further examined in sensitivity analyses, including restricted cubic spline regression, Box-Tidwell test, and log-transformation (**Supplementary Table 11**). Notably, these exploratory analyses suggested a potential non-linear relationship between CA15–3 and ARM lymph node metastasis, which warrants further validation in larger cohorts.

When an ARM node also serves as the sentinel lymph node, it is termed a sentinel ARM lymph node. Our study also identified this anatomical overlap as a significant predictor of ARM lymph node metastasis. Among the 142 patients enrolled, the crossover rate was 19.72% (28/142), which was consistent with previously reported data. A meta-analysis reported a crossover rate of 19.6% (95% CI: 14.4%–26.1%) between SLNs and ARM lymph nodes (42). In another study by Tummel et al. (5), only 18 out of 472 patients (3.8%) undergoing SLNB exhibited overlap between SLNs and ARM lymph nodes; among 213 patients who underwent ALND, the crossover rate was 5.6%. Variability in reported crossover rates across studies may be attributed to technical factors, such as the type of dye used, injection site (peritumoral vs. subareolar), and procedural timing, which might influence lymphatic mapping accuracy and completeness. These findings suggest that even in patients undergoing more extensive axillary dissection, the concurrence of SLN and ARM nodes remains low, further supporting the notion that lymphatic drainage pathways from the breast and the affected upper limb are largely anatomically distinct. Notably, in this study, 50% (14/28) of these sentinel ARM lymph nodes showed metastatic involvement. This finding aligns with previous reports indicating that sentinel ARM lymph node crossover is the primary mechanism underlying ARM node metastasis in patients with SLN-positive breast cancer (43–45). Noguchi et al. demonstrated in a stratified analysis that when sentinel and ARM lymph nodes coincide, the risk of metastasis is significantly higher in these dual-role nodes compared to non-sentinel ARM nodes. Furthermore, among patients with pathologically confirmed SLN metastasis, the residual non-sentinel ARM nodes also exhibited a higher metastatic burden than those in SLN-negative patients. Collectively, these findings underscore that the presence of sentinel ARM lymph nodes is a robust indicator of increased metastatic risk within the ARM pathway (46, 47). Biologically, this heightened risk is consistent with the role of SLNs as the initial drainage site for tumor cells, making them the most vulnerable to early metastatic spread. The co-localization of sentinel and ARM nodes suggests that the upper extremity lymphatic drainage pathway may be directly exposed to tumor dissemination, potentially compromising the oncologic safety of routine ARM node preservation. This anatomical vulnerability highlights the need for careful assessment when considering ARM lymph node preservation.

Beyond nodal identity, morphological features can also reflect metastatic potential, as the presence of metastasis in lymph nodes may affect the consistency, size, and contour of the lymph nodes. Enlargement may reflect tumor infiltration, reactive hyperplasia, or architectural distortion. Significantly, our study identified that ARM lymph node length was an independent predictor of metastasis. This is clinically relevant, as prior evidence supports the safety and benefit of selectively preserving benign-appearing ARM lymph nodes (excluding sentinel ARM lymph nodes) during axillary surgery, which has been associated with a reduced incidence of BCRL (48, 49). Therefore, we propose that intraoperative visualization and measurement of ARM node size using ARM technology may serve as a practical strategy to assess metastatic risk in real time, enabling more informed decisions regarding node preservation versus removal.

Ultrasound (US) remains the primary imaging modality for breast cancer screening and preoperative axillary evaluation (50). Several established sonographic features were associated with metastatic nodes, including cortical thickening (>3 mm), loss of the echogenic fatty hilum, indistinct margins, a long-axis-to-short-axis ratio <2, and peripheral vascularity (51, 52). Due to technical constraints, this study incorporated only four ultrasound parameters: ultrasound grading, posterior echo attenuation, blood flow, and a composite assessment of abnormal lymph nodes. Among these, the presence of ultrasound-detected abnormal lymph nodes emerged as an independent predictor of ARM lymph node metastasis, likely reflecting more advanced nodal disease. This finding aligns with prior studies linking similar ultrasound features to non-sentinel axillary lymph node involvement (53–56). Additionally, although a strong association between pathological nodal stage (pN) and ARM lymph node metastasis has been reported (16, 57), we excluded pN from our model due to its inherent postoperative nature and lack of preoperative predictability.

The developed risk-prediction nomogram incorporates six independent predictors, which are all preoperatively accessible, enhancing its clinical relevance. The resulting nomogram achieved an impressive AUC of 0.934, indicating excellent discriminatory performance. To the best of our knowledge, this is the first study to systematically investigate and model the risk of ARM lymph node metastasis in breast cancer patients. All included variables are readily obtainable in routine clinical practice, enhancing its practicality and potential for widespread clinical application. The nomogram may help identify low-risk patients who could safely undergo selective ARM node preservation, thereby minimizing lymphedema risk without compromising oncologic outcomes.

Although the nomogram demonstrated robust discriminative ability in the development cohort (AUC = 0.934), its performance decreased in the temporal validation cohort (AUC = 0.826), with suboptimal calibration (calibration slope = 0.327; observed-to-expected ratio = 1.295). This calibration drift, where the model tended to underpredict the absolute risk in later patients, suggests that while the nomogram maintains acceptable rank-order discrimination for risk stratification, its absolute probability estimates are less stable. This phenomenon is likely attributable to the limited sample size of the validation cohort and the observed baseline age shift between cohorts, rather than fundamental model misspecification. Nevertheless, these findings underscore the need for external validation in larger, multicenter cohorts and potential model recalibration before clinical implementation.

Several limitations should be acknowledged. First, the retrospective single-center design and limited sample size yielded a low event-per-variable ratio, which may increase the risk of model overfitting, although Firth penalized regression supported the main findings. Second, the temporal validation cohort was derived from the same institution, limiting the strength of external validity. Third, exclusion of patients without identifiable ARM lymph nodes may introduce selection bias. Finally, the underlying biological mechanisms were not investigated, and incorporation of molecular or high-resolution imaging data may further improve predictive performance.

## 5 Conclusion

This study highlights the importance of predicting ARM lymph node metastasis in breast cancer patients to optimize surgical decision-making. We developed a clinically applicable nomogram incorporating clinical, serological, imaging, and intraoperative anatomical predictors. The model demonstrated excellent discrimination in the development cohort and acceptable discrimination in an exploratory temporal validation cohort. However, calibration was suboptimal in the temporal validation cohort, indicating that further multicenter external validation and recalibration are required before routine clinical implementation. This nomogram may help identify patients at low risk of ARM lymph node metastasis who could benefit from selective ARM node preservation, thereby balancing oncologic safety with the reduction of long-term complications such as lymphedema.

## Data Availability

The raw data supporting the conclusions of this article will be made available by the authors, without undue reservation.
