# Supplementary material for: Development and validation of a nomogram for predicting axillary reverse mapping lymph node metastasis in breast cancer patients
Source: Front Oncol. 2026 Jul 21;16:1854483. doi: 10.3389/fonc.2026.1854483 (PMC13433204; doi:10.3389/fonc.2026.1854483)
Supplement: Supplementary file 1 [file DataSheet1.docx]

**Supplementary Figure**

**Figure S1. Patterns of SLNs and ARM lymph nodes identification under different detection modes.** Representative ex vivo specimens are shown under methylene blue (MB) and indocyanine green (ICG) fluorescence modes. (A) Sentinel ARM lymph node and post-SLN. (B) SLN and ARM lymph node, with short distance. (C) SLN and post-sentinel ARM lymph node. (D) SLN and ARM lymph node, with long distance. Blue, yellow, and green arrows indicate SLNs, post-SLNs, and ARM lymph nodes, respectively. Overlapping arrows denote lymphatic drainage overlap, with blue-green overlap representing sentinel ARM lymph nodes and yellow-green overlap representing post-sentinel ARM lymph nodes.
