# Supplementary material for: Development and validation of a nomogram for predicting axillary reverse mapping lymph node metastasis in breast cancer patients
Source: Front Oncol. 2026 Jul 21;16:1854483. doi: 10.3389/fonc.2026.1854483 (PMC13433204; doi:10.3389/fonc.2026.1854483)
Supplement: Supplementary file 2 [file Table1.docx]

**Table S1. Detailed Baseline Characteristics of the Breast Cancer Patients in the Development Cohort.**

| **Characteristics** | **ARM LNM**  **negative group**  **(n = 109)** | | **ARM LNM**  **positive group**  **(n = 33)** | | | ***P*** | |
| --- | --- | --- | --- | --- | --- | --- | --- |
| Hypertension, n(%) |  | |  | | | 1.000 | |
| No | 93 (85.321) | | 28 (84.848) | | |  | |
| Yes | 16 (14.679) | | 5 (15.152) | | |  | |
| Diabetes, n(%) |  | |  | | | 1 | |
| No | 99 (90.826) | | 30 (90.909) | | |  | |
| Yes | 10 (9.174) | | 3 (9.091) | | |  | |
| Heart disease, n(%) |  | |  | | | 0.432 | |
| No | 99 (90.826) | | 32 (96.970) | | |  | |
| Yes | 10 (9.174) | | 1 (3.030) | | |  | |
| Menopause, n(%) |  | |  | | | 0.972 | |
| No | 40 (36.697) | | 12 (36.364) | | |  | |
| Yes | 69 (63.303) | | 21 (63.636) | | |  | |
| Tumor-areola distance, n(%) |  | |  | | | 0.539 | |
| ≤2cm | 66 (60.550) | | 18 (54.545) | | |  | |
| ＞2cm | 43 (39.450) | | 15 (45.455) | | |  | |
| Nipple inversion, n(%) |  | |  | | | **0.038** | |
| No | 107 (98.165) | | 29 (87.879) | | |  | |
| Yes | 2 (1.835) | | 4 (12.121) | | |  | |
| cT, n(%) |  | |  | | | **0.011** | |
| 1 | 47 (43.119) | | 6 (18.182) | | |  | |
| 2 | 59 (54.128) | | 25 (75.758) | | |  | |
| 3 | 1 (0.917) | | 2 (6.061) | | |  | |
| 4 | 2 (1.835) | | 0 (0.00) | | |  | |
| cN, n(%) |  | |  | | | 0.053 | |
| 0 | 53 (48.624) | | 9 (27.273) | | |  | |
| 1 | 50 (45.872) | | 20 (60.606) | | |  | |
| 2 | 4 (3.670) | | 4 (12.121) | | |  | |
| 3 | 2 (1.835) | | 0 (0.00) | | |  | |
| RBC, Mean ± SD | | 4.304 ± 0.360 | | 4.395 ± 0.317 | 0.192 | |  |
| WBC, Mean ± SD | | 5.881 ± 1.460 | | 5.597 ± 1.228 | 0.313 | |  |
| Neutrophil count,  Mean ± SD | 3.424 ± 1.155 | | 3.302 ± 0.884 | | | 0.575 | |
| Lymphocyte count,  Mean ± SD | 1.915 ± 0.537 | | 1.805 ± 0.596 | | | 0.318 | |
| Platelet count, Mean ± SD | 258.830 ± 62.947 | | 260.394 ± 53.615 | | | 0.897 | |
| Monocyte count,  Mean ± SD | 0.394 ± 0.013 | | 0.399 ± 0.106 | | | 0.789 | |
| HDL(U/L), Mean ± SD | 1.368 ± 0.255 | | 1.338 ± 0.257 | | | 0.567 | |
| LDL(U/L), Mean ± SD | 3.003 ± 0.886 | | 2.962 ± 0.777 | | | 0.810 | |
| Luteinizing hormone(U/L),  Mean ± SD | | 23.023 ± 15.600 | | 19.768 ± 16.662 | 0.303 | |  |
| Testosterone(U/L),  Mean ± SD | | 0.785 ± 0.487 | | 0.650 ± 0.403 | 0.149 | |  |
| Prolactin (U/L), M (Q₁, Q₃) | | 257.300 (169.600, 383.900) | | 253.900 (181.500, 362.100) | 0.852 | |  |
| Follicle Stimulating Hormone(U/L), M (Q₁, Q₃) | | 36.720 (8.430, 58.980) | | 33.180 (6.450, 60.390) | 0.496 | |  |
| Progesterone(U/L),  M (Q₁, Q₃) | | 0.450 (0.240, 0.900) | | 0.600 (0.320, 1.580) | 0.077 | |  |
| Estradiol(U/L), M (Q₁, Q₃) | | 51.090 (16.000, 256.800) | | 78.810 (16.000, 246.700) | 0.686 | |  |
| NLR, M (Q₁, Q₃) | | 1.694 (1.392, 2.298) | | 1.870 (1.442, 2.320) | 0.390 | |  |
| Pathological tumor long diameter(cm)（cm）, M (Q₁, Q₃) | 2.000 (1.500, 2.800) | | 2.500 (2.000, 3.500) | | | **0.021** | |
| Histological grade, n(%) |  | |  | | | 0.616 | |
| 1 | 5 (4.587) | | 1 (3.030) | | |  | |
| 2 | 64 (58.716) | | 24 (72.727) | | |  | |
| 3 | 35 (32.110) | | 7 (21.212) | | |  | |
| 4 | 5 (4.587) | | 1 (3.030) | | |  | |
| Invasive carcinoma with in situ, n(%) |  | |  | | | 0.587 | |
| No | 47 (43.119) | | 16 (48.485) | | |  | |
| Yes | 62 (56.881) | | 17 (51.515) | | |  | |
| Special cancer status, n(%) |  | |  | | | 0.152 | |
| No | 94 (86.239) | | 25 (75.758) | | |  | |
| Yes | 15 (13.761) | | 8 (24.242) | | |  | |
| Tumor count, n(%) |  | |  | | | 1 | |
| Monofocal | 97 (88.991) | | 30 (90.909) | | |  | |
| multifocal | 12 (11.009) | | 3 (9.091) | | |  | |
| Nipple invasion, n(%) |  | |  | | | 0.716 | |
| No | 106 (97.248) | | 31 (93.939) | | |  | |
| Yes | 3 (2.752) | | 2 (6.061) | | |  | |
| Latissimus dorsi variation, n(%) |  | |  | | | 0.512 | |
| No | 92 (84.404) | | 30 (90.909) | | |  | |
| Yes | 17 (15.596) | | 3 (9.091) | | |  | |
| pT, n(%) |  | |  | | | 0.582 | |
| 1 | 53 (48.624) | | 13 (39.394) | | |  | |
| 2 | 53 (48.624) | | 19 (57.576) | | |  | |
| 3 | 2 (1.835) | | 1 (3.030) | | |  | |
| 4 | 1 (0.917) | | 0 (0.00) | | |  | |
| pN, n(%) |  | |  | | | **<.001** | |
| 0 | 56 (51.376) | | 0 (0.00) | | |  | |
| 1 | 35 (32.110) | | 10 (30.303) | | |  | |
| 2 | 17 (15.596) | | 14 (42.424) | | |  | |
| 3 | 1 (0.917) | | 9 (27.273) | | |  | |
| ER, n(%) |  | |  | | | 0.095 | |
| 0 | 26 (23.853) | | 2 (6.061) | | |  | |
| 1+ | 25 (22.936) | | 12 (36.364) | | |  | |
| 2+ | 38 (34.862) | | 14 (42.424) | | |  | |
| 3+ | 20 (18.349) | | 5 (15.152) | | |  | |
| PR, n(%) |  | |  | | | **<.001** | |
| 0+ | 35 (32.110) | | 3 (9.091) | | |  | |
| 1+ | 15 (13.761) | | 17 (51.515) | | |  | |
| 2+ | 32 (29.358) | | 12 (26.364) | | |  | |
| 3+ | 27 (24.771) | | 1 (3.030) | | |  | |
| HER2, n(%) |  | |  | | | 0.578 | |
| 0 | 35 (32.110) | | 7 (21.212) | | |  | |
| 1+ | 18 (16.514) | | 8 (24.242) | | |  | |
| 2+ | 29 (26.606) | | 10 (30.303) | | |  | |
| 3+ | 27 (24.771) | | 8 (24.242) | | |  | |
| HER2 positive, n(%) |  | |  | | | 0.753 | |
| No | 83 (76.147) | | 26 (78.788) | | |  | |
| Yes | 26 (23.853) | | 7 (21.212) | | |  | |
| Ki67 level, n(%) |  | |  | | | **<.001** | |
| Low | 54 (49.541) | | 0 (0.00) | | |  | |
| High | 55 (50.459) | | 33 (100.000) | | |  | |
| Ultrasound attenuation, n(%) |  | |  | | | 0.356 | |
| No | 66 (60.550) | | 17 (51.515) | | |  | |
| Yes | 43 (39.450) | | 16 (48.485) | | |  | |
| Ultrasound blood flow, n(%) |  | |  | | | 0.376 | |
| No | 28 (25.688) | | 6 (18.182) | | |  | |
| Yes | 81 (74.312) | | 27 (81.818) | | |  | |

Abbreviations: SD: standard deviation, M: Median, Q₁: 1st Quartile, Q₃: 3rd Quartile; BMI, Body Mass Index; RBC, Red Blood Cell Count; WBC, White Blood Cell Count; HDL, High-Density Lipoprotein; LDL, Low-Density Lipoprotein; ER, Estrogen Receptor; PR, Progesterone Receptor; HER2, Human Epidermal Growth Factor Receptor 2; Ki67, Ki67 Proliferation Index.
