# Supplementary material for: Development and validation of a nomogram for predicting axillary reverse mapping lymph node metastasis in breast cancer patients
Source: Front Oncol. 2026 Jul 21;16:1854483. doi: 10.3389/fonc.2026.1854483 (PMC13433204; doi:10.3389/fonc.2026.1854483)
Supplement: Supplementary file 3 [file Table10.docx]

**Table S10. Multicollinearity assessment of predictors included in the final nomogram.**

| **A. Variance inflation factor analysis** | | | | | |  |
| --- | --- | --- | --- | --- | --- | --- |
| **Predictor** | | | | | **VIF** |  |
| Age at menarche ≤12 years | | | | | 1.077 |  |
| Fibrinogen | | | | | 1.08 |  |
| CA15-3 | | | | | 1.049 |  |
| PLR | | | | | 1.078 |  |
| ARM lymph node long diameter | | | | | 1.133 |  |
| Sentinel ARM lymph node | | | | | 1.124 |  |
| Ultrasound-detected abnormal lymph nodes | | | | | 1.077 |  |
| **B. Spearman correlation matrix for continuous predictors** | | | | | | |
| **Variable** | **Fibrinogen** | **CA15-3** | **PLR** | **ARM lymph node long diameter** | | |
| Fibrinogen | 1 | 0.162 | -0.033 | 0.169 | | |
| CA15-3 | 0.162 | 1 | 0.092 | 0.017 | | |
| PLR | -0.033 | 0.092 | 1 | -0.089 | | |
| ARM lymph node long diameter | 0.169 | 0.017 | -0.089 | 1 | | |
