# Supplementary material for: Development and validation of a nomogram for predicting axillary reverse mapping lymph node metastasis in breast cancer patients
Source: Front Oncol. 2026 Jul 21;16:1854483. doi: 10.3389/fonc.2026.1854483 (PMC13433204; doi:10.3389/fonc.2026.1854483)
Supplement: Supplementary file 4 [file Table11.docx]

| **Table S11. Linearity assessment and functional form evaluation of CA15-3.** | | |
| --- | --- | --- |
| **Analysis** | **Result** | **Interpretation** |
| RCS overall association | P = 0.0050 | CA15-3 was associated with ARM LNM |
| RCS non-linear component | P = 0.0145 | Evidence of non-linearity |
| Box-Tidwell test | P = 0.0146 | Linearity assumption not fully satisfied |
| CA15-3 >30 U/mL distribution | 3 above cut-off, 0 events | Cut-off not informative |
| Log-transformed CA15-3 model | AIC = 95.281, AUC = 0.925 | Preferred functional form |
