# Supplementary material for: Development and validation of a nomogram for predicting axillary reverse mapping lymph node metastasis in breast cancer patients
Source: Front Oncol. 2026 Jul 21;16:1854483. doi: 10.3389/fonc.2026.1854483 (PMC13433204; doi:10.3389/fonc.2026.1854483)
Supplement: Supplementary file 5 [file Table2.docx]

| **LN situation** | **Total**  **(n=142)** | | **ARM LNM negative group**  **(n=109)** | | **ARM LNM**  **positive group**  **(n=33)** | **Statistic** | ***P*** |
| --- | --- | --- | --- | --- | --- | --- | --- |
| Sentinel lymph node count,  M(Q₁, Q₃) | | 2.000 (1.000, 3.000) | | 2.000 (1.000, 3.000) | 2.000 (1.000, 3.000) | Z=-0.298 | 0.766 |
| Total ARM lymph nodes,  M(Q₁, Q₃) | | 3.000 (1.000, 5.000) | | 3.000 (1.000, 5.000) | 4.000 (2.000, 6.000) | Z=-2.025 | **0.043** |
| Group I ARM lymph nodes,  M(Q₁, Q₃) | | 2.000 (1.000, 4.000) | | 2.000 (1.000, 4.000) | 3.000 (2.000, 5.000) | Z=-1.767 | 0.077 |
| ARM lymph node long diameter, M(Q₁, Q₃) | | 1.500 (1.200, 2.200) | | 1.500 (1.000, 2.000) | 2.400 (1.500, 3.000) | Z=-4.180 | **<.001** |
| Sentinel lymph node metastasis, n(%) | |  | |  |  | χ²=32.625 | **<.001** |
| No | | 66 (46.479) | | 65 (59.633) | 1 (3.030) |  |  |
| Yes | | 76 (53.521) | | 44 (40.367) | 32 (96.970) |  |  |
| Parasentinel Lymph Node, n(%) | |  | |  |  | χ²=0.467 | 0.494 |
| No | | 110 (77.465) | | 83 (76.147) | 27 (81.818) |  |  |
| Yes | | 32 (22.535) | | 26 (23.853) | 6 (18.182) |  |  |
| Postsentinel lymph node, n(%) | |  | |  |  | χ²=0.066 | 0.798 |
| No | | 79 (55.634) | | 60 (55.046) | 19 (57.576) |  |  |
| Yes | | 63 (44.366) | | 49 (44.954) | 14 (42.424) |  |  |
| Parasentinel Lymph Node Metastasis Status, n(%) | |  | |  |  | χ²=12.582 | **0.002** |
| No | | 26 (18.310) | | 25 (22.936) | 1 (3.030) |  |  |
| Yes | | 8 (5.634) | | 3 (2.752) | 5 (15.152) |  |  |
| Unknow | | 108 (76.056) | | 81 (74.312) | 27 (81.818) |  |  |
| Postsentinel lymph node metastasis status, n(%) | |  | |  |  | χ²=21.599 | **<.001** |
| No | | 42 (29.577) | | 40 (36.697) | 2 (6.061) |  |  |
| Yes | | 21 (14.789) | | 9 (8.257) | 12 (36.364) |  |  |
| Unknow | | 79 (55.634) | | 60 (55.046) | 19 (57.576) |  |  |
| Sentinel ARM lymph node, n(%) | |  | |  |  | χ²=14.001 | **<.001** |
| No | | 114 (80.282) | | 95 (87.156) | 19 (63.636) |  |  |
| Yes | | 28 (19.718) | | 14 (12.844) | 14 (36.364) |  |  |
| Common stem status, n(%) | |  | |  |  | χ²=20.936 | **<.001** |
| No | | 87 (61.268) | | 78 (71.560) | 9 (27.273) |  |  |
| Yes | | 55 (38.732) | | 31 (28.440) | 24 (72.727) |  |  |

**Table S2. Lymph nodes situation of Breast Cancer Patients Stratified by ARM Lymph Node Metastasis Status**
