# Supplementary material for: Development and validation of a nomogram for predicting axillary reverse mapping lymph node metastasis in breast cancer patients
Source: Front Oncol. 2026 Jul 21;16:1854483. doi: 10.3389/fonc.2026.1854483 (PMC13433204; doi:10.3389/fonc.2026.1854483)
Supplement: Supplementary file 6 [file Table3.docx]

| **Distance between ARM LNs and SLNs** | **n=142（%）** |
| --- | --- |
| D1（0cm） | 28（19.72） |
| D2（>0 and ≤1 cm） | 2（1.41） |
| D3（>1 and ≤2 cm） | 15（10.56） |
| D4（>2 and ≤3 cm） | 19（13.38） |
| D5（>3 and ≤4 cm） | 27（19.01） |
| D6（>4 and ≤5 cm） | 23（16.19） |
| D7（＞5cm） | 25（17.61） |

**Table S3. Distribution of distance between ARM LNs and SLNs**
