# Supplementary material for: Development and validation of a nomogram for predicting axillary reverse mapping lymph node metastasis in breast cancer patients
Source: Front Oncol. 2026 Jul 21;16:1854483. doi: 10.3389/fonc.2026.1854483 (PMC13433204; doi:10.3389/fonc.2026.1854483)
Supplement: Supplementary file 7 [file Table4.docx]

**Table S4. Axillary lymph drainage distribution pattern.**

| **Distance** | | **Common stem status** | | **Total Number (%)** |
| --- | --- | --- | --- | --- |
|  |  | **Common stem, n (%)** | **No common stem, n (%)** |  |
| **Close type, n (%)** | | 32 (22.54) | 11 (7.74) | 43 (30.28) |
| **Distant type, n (%)** | | 23 (16.20) | 76 (53.52) | 99 (69.71) |
| **Total Number (%)** | | 55 (38.73) | 87 (61.27) | 142 (100) |
|  |  |  |  |  |
