# Supplementary material for: Development and validation of a nomogram for predicting axillary reverse mapping lymph node metastasis in breast cancer patients
Source: Front Oncol. 2026 Jul 21;16:1854483. doi: 10.3389/fonc.2026.1854483 (PMC13433204; doi:10.3389/fonc.2026.1854483)
Supplement: Supplementary file 8 [file Table6.docx]

**Table S6. Baseline characteristics of the development and temporal validation cohorts.**

| **Variable** | **Development cohort**  **(n=142)** | | **Temporal validation cohort**  **(n=54)** | | **P** |
| --- | --- | --- | --- | --- | --- |
| No. of patients | 142 | | 54 | |  |
| Age, years | 53.00 (47.00, 60.00) | | 49.00 (40.25, 56.75) | | **0.023** |
| BMI, kg/m² | 24.35 (22.76, 26.72) | | 24.97 (23.10, 26.90) | | 0.572 |
| Fibrinogen, g/L | 2.93 (2.58, 3.25) | | 2.94 (2.64, 3.19) | | 0.875 |
| CA153, U/mL | 10.86 (7.61, 14.45) | | 12.85 (8.02, 15.88) | | 0.066 |
| Platelet-to-lymphocyte ratio | 142.35 (106.47, 170.18) | | 127.11 (92.33, 173.99) | | 0.278 |
| ARM lymph node long diameter, cm | 1.50 (1.20, 2.20) | | 1.50 (1.10, 2.00) | | 0.627 |
| Age at menarche |  |  | |  | |
| ≤12 years | 21 (14.8%) | | 8 (14.8%) | | 1.000 |
| >12 years | 121 (85.2%) | | 46 (85.2%) | |  |
| Sentinel ARM lymph node |  |  | |  | |
| No | 114 (80.3%) | | 46 (85.2%) | | 0.558 |
| Yes | 28 (19.7%) | | 8 (14.8%) | |  |
| Ultrasound-detected abnormal lymph nodes |  |  | |  | |
| No | 79 (55.6%) | | 32 (59.3%) | | 0.767 |
| Yes | 63 (44.4%) | | 22 (40.7%) | | |
| ARM lymph node metastasis |  | |  | |  |
| No | 109 (76.8%) | | 38 (70.4%) | | 0.460 |
| Yes | 33 (23.2%) | | 16 (29.6%) | | |

Abbreviations: ARM, axillary reverse mapping; BMI, body mass index; CA15-3, cancer antigen 153.
