# Supplementary material for: Development and validation of a nomogram for predicting axillary reverse mapping lymph node metastasis in breast cancer patients
Source: Front Oncol. 2026 Jul 21;16:1854483. doi: 10.3389/fonc.2026.1854483 (PMC13433204; doi:10.3389/fonc.2026.1854483)
Supplement: Supplementary file 9 [file Table7.docx]

**Table S7. Predictive performance of the nomogram in the development and temporal validation cohorts.**

| **Metric** | **Development cohort (n=142)** | **Temporal validation cohort (n=54)** |
| --- | --- | --- |
| Sample size, n | 142 | 54 |
| ARM lymph node metastasis events, n (%) | 33 (23.2%) | 16 (29.6%) |
| Non-events, n | 109 | 38 |
| AUC (95% CI) | 0.934 (0.892–0.977) | 0.826 (0.708–0.943) |
| Cutoff | 0.115 | 0.115 |
| True positives, n | 32 | 13 |
| True negatives, n | 82 | 29 |
| False positives, n | 27 | 9 |
| False negatives, n | 1 | 3 |
| Sensitivity | 0.970 | 0.812 |
| Specificity | 0.752 | 0.763 |
| Accuracy | 0.803 | 0.778 |
| Positive predictive value | 0.542 | 0.591 |
| Negative predictive value | 0.988 | 0.906 |
| Brier score | 0.082 | 0.168 |
| Observed events, n | 33 | 16 |
| Expected events, n | 33.000 | 12.351 |
| Observed-to-expected ratio | 1.000 | 1.295 |
| Calibration intercept | 0.000 | 0.786 |
| Calibration slope | 1.000 | 0.327 |

Abbreviations: AUC, area under the receiver operating characteristic curve; CI, confidence interval; PPV, positive predictive value; NPV, negative predictive value.
