# Supplementary material for: Development and validation of a nomogram for predicting axillary reverse mapping lymph node metastasis in breast cancer patients
Source: Front Oncol. 2026 Jul 21;16:1854483. doi: 10.3389/fonc.2026.1854483 (PMC13433204; doi:10.3389/fonc.2026.1854483)
Supplement: Supplementary file 10 [file Table8.docx]

**Table S8. Comparison between conventional logistic regression and Firth penalized logistic regression**

| Variable | Conventional logistic regression | | Firth penalized logistic regression | |
| --- | --- | --- | --- | --- |
|  | OR (95% CI) | **P** | OR (95% CI) | **P** |
| Menarche age group |  |  |  |  |
| ≤12 | Reference | | Reference | |
| ＞12 | 0.056 (0.009–0.264) | <0.001 | 0.070 (0.013–0.306) | <0.001 |
| Fibrinogen | 5.860 (1.455–23.605) | 0.013 | 4.642 (1.383–18.630) | 0.012 |
| CA15-3 | 1.155 (1.061–1.256) | <0.001 | 1.136 (1.055–1.233) | 0.001 |
| PLR | 1.016 (1.005–1.027) | 0.004 | 1.014 (1.005–1.025) | 0.003 |
| Sentinel ARM lymph node |  |  |  |  |
| No | Reference | | Reference | |
| Yes | 4.148 (0.913–18.836) | 0.065 | 3.550 (0.876–15.375) | 0.076 |
| ARM lymph node long diameter | 4.574 (1.971–10.614) | <0.001 | 3.815 (1.865–8.936) | <0.001 |
| Ultrasound abnormal lymph nodes |  | |  | |
| No | Reference | | Reference | |
| Yes | 11.474 (2.584–50.954) | <0.001 | 8.462 (2.202–38.336) | <0.001 |

ARM, axillary reverse mapping; CA15-3, cancer antigen 15-3; PLR, platelet-to-lymphocyte ratio; OR, odds ratio; CI, confidence interval.
