# Supplementary material for: Development and validation of a nomogram for predicting axillary reverse mapping lymph node metastasis in breast cancer patients
Source: Front Oncol. 2026 Jul 21;16:1854483. doi: 10.3389/fonc.2026.1854483 (PMC13433204; doi:10.3389/fonc.2026.1854483)
Supplement: Supplementary file 11 [file Table9.docx]

**Table S9. Performance comparison between the original and simplified models.**

| **Model** | **Cohort** | **N** | **Events** | **AUC (95% CI)** | **Brier score** | **OE ratio** | **Calibration intercept** | **Calibration slope** |
| --- | --- | --- | --- | --- | --- | --- | --- | --- |
| Original 7-predictor model | Development cohort | 142 | 33 | 0.934 (0.892–0.977) | 0.082 | 1 | 0 | 1 |
| Original 7-predictor model | Temporal validation cohort | 54 | 16 | 0.826 (0.708–0.943) | 0.168 | 1.295 | 0.786 | 0.327 |
| AIC-selected model | Development cohort | 142 | 33 | 0.934 (0.892–0.977) | 0.082 | 1 | 0 | 1 |
| AIC-selected model | Temporal validation cohort | 54 | 16 | 0.826 (0.708–0.943) | 0.168 | 1.295 | 0.786 | 0.327 |
| BIC-selected model | Development cohort | 142 | 33 | 0.925 (0.871–0.980) | 0.08 | 1 | 0 | 1 |
| BIC-selected model | Temporal validation cohort | 54 | 16 | 0.822 (0.704–0.941) | 0.166 | 1.282 | 0.737 | 0.332 |

The AIC-selected model was identical to the original seven-predictor model. The BIC-selected model removed sentinel ARM lymph node status. Both simplified models were developed in the development cohort and then directly applied to the temporal validation cohort without coefficient re-estimation, variable re-selection, or cutoff optimization. Abbreviations: AIC, Akaike information criterion; BIC, Bayesian information criterion; AUC, area under the receiver operating characteristic curve; CI, confidence interval; O/E ratio, observed-to-expected event ratio.
